# Seasonal Variation of Agility, Speed and Endurance Performance in Young Elite Soccer Players

**DOI:** 10.3390/sports5010012

**Published:** 2017-02-04

**Authors:** Michal Dragijsky, Tomas Maly, Frantisek Zahalka, Egon Kunzmann, Mikulas Hank

**Affiliations:** Sport Research Center, Faculty of Physical Education and Sport, Charles University in Prague, Prague 162 52, Czech Republic; dragijsky@gmail.com (M.D.); zahalka@ftvs.cuni.cz (F.Z.); egonkunzmann@atlas.cz (E.K.); miki.hank@gmail.com (M.H.)

**Keywords:** motor skills, soccer performance, talent identification, football, training and testing

## Abstract

The aim of this study was to investigate changes in the linear running speed (LRS) for 30 m, change of direction speed (CODS), and endurance in young elite Czech soccer players. The following tests were conducted to assess CODS and endurance: Agility 505 turning toward the dominant (A505DL) and non-dominant lower limb (A505NL); Illinois Agility Test (IAT); and intermittent test (Yo-Yo IRT1). During the soccer season, we investigated performance at the following time periods: the start (t_1_) and the end of the pre-season period (t_2_); during (t_3_) and at the end of the competitive period (t_4_). Repeated measurement analysis of variance revealed a significant effect of time period on selected fitness parameters (p < 0.05). Post hoc analysis for test A505DL revealed significant improvements of performance at t_3_ (2.71 ± 0.08 s) and t_4_ (2.72 ± 0.06 s) compared to t_1_ (2.81 ± 0.09 s). A505NL was significantly different between t_1_ (2.83 ± 0.09 s) and t_2_ (2.76 ± 0.09 s), t_3_ (2.7 ± 0.07 s) and t_4_ (2.71 ± 0.09 s). Performance of CODS at t_1_ for the IAT (18.82 ± 0.56 s) was significantly lower (p < 0.05) than any other time period (t_2_ = 18.52 ± 0.63 s, t_3_ = 17.94 ± 0.51 s, t_4_ = 17.89 ± 0.66 s). The power of LRS was significantly different at t_3_ (4.99 ± 0.15 s), and t_4_ (4.98 ± 0.17 s) compared to t_1_ (5.15 ± 0.21 s), and t_2_ (5.07 ± 0.14 s). For the Yo-Yo IRT1 test, we observed a significant increase in performance between t_1_ (625.26 ± 170.34 m), t_2_ (858.95 ± 210.55 m), and t_3_ (953.68 ± 229.88 m). These results show the impact of soccer season time period on young soccer player performance and may further serve as a basis for comparison with similar research conducted by peers. These results may aid sports practice for clinicians, conditioning coaches, soccer coaches and physiotherapeutic coaches.

## 1. Introduction

In soccer, there are demands imposed on soccer players in terms of fitness readiness requirements to produce power, explosiveness, speed, agility, balance, body stability, flexibility and an adequate level of endurance [1,2,3]. Maintaining a high level of these components throughout the season is necessary for achieving consistent high-quality performance, while the basis for these individual components of players is built during youth. Aerobic capacity is an important factor which, in addition to the quality of the game itself, ultimately affects the final position of the teams in the league [4,5]. Moreover, aerobic capacity has beneficial effects on parameters such as total time spent on high intensity activities during the game, number of sprints and the number of contacts with the ball during the match. High aerobic capacity also enhances recovery from high-intensity interval loads [6,7]. Components of anaerobic capacity have the biggest impact on the final outcome of the match and are characterized by repeated short high-intensity activity. The number of these high-intensity activities during the match falls in the range of 150–250 [8] and include acceleration, maximum speed and agility [9]. It is essential to stimulate and maintain these skills constantly during the training process. 

High-intensity physical activities are similar in terms of morphological and biochemical determinants and are believed to be interconnected [10], however they are affected by the method of operation and player position. During the game, players carry out more than 1200 acyclic or unforeseen changes in direction in various movement intensities [11]. Short sprint, acceleration, deceleration, turning, jump kicks into the ball, and stopping an opponent are purposeful and dangerous physical activities during the game [12,13] and are dependent on the explosiveness and the level of muscular strength of the player [14,15]. The level and balance between anaerobic and aerobic capacity is an important parameter for high performance [16,17].

Previous studies dealing with variability of performance during the season have been focused on professional [18,19] and semi-professional adult players [20] and one study of young players focused on changes in power skills and repeated sprint ability [21]. In the present study, we focused on young players from the under 13 years-of-age (U13) category where we observed changes over time from pre-season preparation to the end of the competitive period. Monitoring of physical preparedness parameters in the youth age group is an important part of the training process because it is an important period of physical development. Sport coaches and scientists can analyse the factors that affect athletic performance (health, genetic predisposition or how the player works in the training process) to provide useful information about the strengths and weaknesses of young soccer players.

The aim of this study was to investigate changes in performance of linear running speed, change of direction speed and endurance throughout the season of young soccer players.

## 2. Materials and Methods

### 2.1. Participants

The sample consisted of 28 players from the U13 category of the highest level Czech league during the 2015–2016 season (age: 11.7 ± 0.5 ages, body height: 149.3 ± 7.8 cm, body weight: 40.5 ± 5.7 kg). Players completed five trainings and one match during the week microcycle. The average soccer experience of players was 5.59 ± 0.89 years. During testing, all subjects were healthy, and none was recently injured.

### 2.2. Time Management of Research

The pre-season period started 20 January and ran until 28 March. There were 69 training units (TU) and 10 matches (M) during this period. The first half of the competitive period lasted from 28 March until 9 May with 23 TU and nine M. The second half of the competitive period was from 9 May until 20 June and included 23 TU and 11 M. 

**Figure 1:**
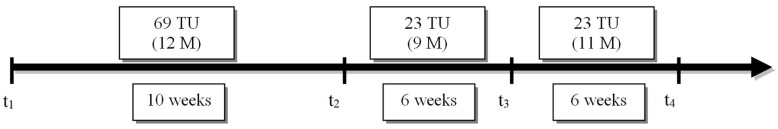
Schematic representation of the study design. Summarization of training units (TU) and number of matches (M) during the pre-season period (t_1_–t_2_), first half of the competitive period (t_2_–t_3_) and second half of the competitive period (t_3_–t_4_).

Individual tests were conducted four times during the season on the surface of fourth generation artificial grass at the time of regular afternoon workouts (14:30 to 15:30 P.M.). All the tests were performed wearing football boots. A controlled warm-up by the trainer that lasted 25 minutes and consisted of jogging (5 min), static stretching (5 min), dynamic stretching (6 min), athletic alphabet (5 min) and four runs (40 m with speed change), occurred before testing. Tests we carried out during the spring season of 2015–2016. Testing occurred at the beginning of the pre-season training period (t_1_), the start of the competitive period (t_2_), the middle of the competitive period (t_3_) and the end of the competitive period (t_4_) (Figure 1). The day before each test, players underwent a light training session. 

No subjects performing physical testing had undergone any surgery on the knee joint, and within two days prior to testing, they had not undergone any exhausting physical activity. All test subjects were notified of the content and implementation of testing procedures and consent was obtained by signatures from their parents. This research was approved by the ethical committee of the Faculty of Physical Education and Sport, Charles University in Prague (Czech Republic). Measurements were carried out in accordance with ethical standards of the Declaration of Helsinki and ethical standards in sport and exercise science research [22].

### 2.3. Process of Physical Performance Assessment

#### 2.3.1. Agility 505 Test (A505)

The ability to perform single direction changes was investigated using the Agility 505 test, turning towards the dominant (A505DL) and non-dominant (A505NL) lower limb [14]. On each side, players completed two trials.

#### 2.3.2. Illinois Agility Test (IAT)

The ability to perform repeated quick direction changes was evaluated through the Illinois agility test (IAT) [23]. Players began the test on their own impulse from a standing position. Each of the players completed the test two times and for the analysis, we included the attempt with the better time.

#### 2.3.3. Linear Running Speed (LRS)

Linear running speed was measured by running 30 m from a standing position. Running time was recorded using two pairs of photocells (Brower Timing System, Utah). Players initiated the start on their own impulse. Players had two attempts and the better performance was kept for analysis.

#### 2.3.4. Intermittent Yo-Yo Recovery Test Level 1(Yo-Yo IRT1)

Endurance was investigated using the Yo-Yo intermittent recovery test level 1 (Yo-Yo IRT1), which is considered a valid and reliable tool for evaluating specific intermittent endurance in soccer [24]. The Yo-Yo IRT1 consists of repeated 2 × 20 m runs back and forth between the start, turn, and finish line at a progressively increased speed controlled by audio bleeps from a tape recorder. Between each running bout, the subjects have a 10-s active rest period, consisting of 2 × 5 m of jogging. If the subjects failed twice to reach the finish line in time, the distance covered is recorded. In the present study, we used the Yo-Yo intermittent recovery test, level 1, which consists of four running bouts at 10–13 km·h^−1^ (0–160 m) and another seven runs at 13.5–14 km·h^−1^ (160–440 m); this continues with stepwise 0.5 km·h^−1^ speed increments after every eight running bouts (i.e., 760, 1080, 1400, 1720 m, etc.) until exhaustion. The test was performed on outdoor running lanes, marked by cones, with a width of 2 m and a length of 20 m. Another cone, placed 5 m behind the finish line, marked the running distance during the active recovery period. All subjects were familiarized with the test from at least one pre-test.

### 2.4. Statistical Analysis

For statistical processing of the data, we used descriptive statistics. Measurements were expressed using the arithmetic mean and the measure of variability was expressed using the standard deviation. Parametric procedures were chosen after the verification of normality of the data distribution using the Shapiro–Wilk test. To assess the significance of the independent variables (seasonal variability) on the dependent variables (A505DL, A505NL, IAT, LRS, Yo-Yo IRT1), a repeated measures analysis of variances (RM ANOVA) was used. To evaluate the sphericity, an assumption of RM ANOVA, a Mauchly’s test was performed. To compare measurements among many variables, we used a Bonferroni post hoc test. We rejected the null hypothesis at the *p* ≤ 0.05 level. Effect size was assessed using the ”Eta square” coefficient (*η^2^*), which explains the proportion of variance of the factor. Effect size was examined as follows: *η^2^* = 0.20 is considered a small effect, *η^2^* = 0.50 is considered a medium effect, and *η^2^* = 0.80 is considered a large effect [25].

Statistical analyses were carried out using IBM^®^ SPSS^®^ v21 (Statistical Package for Social Sciences, Inc., Chicago, IL, USA, 2012). 

## 3. Results

The results of carried out analyses are presented in Table 1. It was observed that the differences in some tests were statistically significant. In the agility 505 test, turning toward the dominant lower limb, players achieved a significant improvement from the start of pre-season (t_1_) to performance during the competitive period (t_3_) (3.6%), and at the end of the competitive period (t_4_) (3.2%). In the same test, turning towards the non-dominant lower limb, we found a significant difference between the first time period (t_1_) and all other measurements (Table 1).

For this test, we also observed significant improvement in the performance from the end of the pre-season period (t_2_) to measurements during the competitive period (t_3_) (2.2%). Similar results were found using the Illinois agility test where performance from the pre-season period (t_1_ = 18.82 ± 0.56 s) was significantly lower compared to the performance from other time periods (t_2_, t_3_, t_4_). Post hoc analysis also revealed a significantly lower time at the end of the pre-season training period (t_2_ = 18.52 ± 0.63 s) compared to that during the competitive period (t_3_ = 17.94 ± 0.51 s), or at the end of the competitive period (t_4_ = 17.89 ± 0.66 s). Pre-season performance (t_1_) was not significantly different for the linear running speed at 30 m distance (*p* > 0.05) compared to the start of the competitive period (t_2_). However, for the competitive periods (t_3_) and (t_4_), there was a significant improvement compared to the start (t_1_) and end of the pre-season period (t_2_).

The pre-season period had the highest effect on players’ endurance (Yo-Yo IRT1), where players managed to increase the efficiency by 27.2% (*p* < 0.01). At time period (t_3_), there was further improvement of this parameter by 9.9% (Table 1).

## 4. Discussion

The aim of this study was to investigate changes in performance of linear running speed, single and multiple direction changes, and endurance during the soccer season in young soccer players from the U13 category. 

Changes in performance of linear running speed over time have been considered by several authors. Significant effects (*p* < 0.001) of the pre-season period on maximum speed during a test run of 50 m with adult soccer players was found by researchers [26]. Authors note a more than 5.13% improvement in maximum running speed. In contrast, the effect of a pre-season training period in our study showed no significant difference in linear running speed at the start (t_1_ = 5.15 ± 0.21 s) and end of the pre-season training period (t_2_ = 5.07 ± 0.14 s). This may be explained by the fact that this component was not particularly stimulated during training sessions. Game aspects, movement games and quick change of direction speed in a small space were emphasized during training. Caldwell and Peters [20] found significantly slower measures in semi-professional players for 15 m sprints in the middle of the season (2.44 ± 0.10 s) than at the beginning of the competitive season (2.49 ± 0.10 s), which is in contrast to our results where players significantly improved compared to the start of competitive period (t_2_ = 5.07 ± 0.14 s, t_3_ = 4.99 ± 0.15 s). These differences may indicate a different adaptation to the training load during the training period between adults and young soccer players. A significant difference was also shown between the time period t_1_ (5.07 ± 0.14 s), and t_4_ (4.98 ± 0.17 s), which may be due to the higher number of adaptation initiatives (more matches) during the competitive period, or morphological changes in the player´s body (increased body height, greater percentage of lean body mass), which could positively affect a player´s motor skills (extended walking pace, increased production of lower extremity muscle strength, etc.). Ostojic [27] found significant improvement (12.6 ± 3.3 %) in the linear running speed of 50 m for major league players. Significant improvement (11.7 ± 2.68 %) in linear running speed for 35 m during the competitive period was observed in Greek adult major league players [17]. Aziz et al. [16] reported significantly better running results for 20 m in Singaporean adults at the end of the competitive period (2.95 ± 0.09 s) compared to the start of the competitive period (3.01 ± 0.10 s); these authors suggest that the training programme and load during the season are responsible. Moreover, it was confirmed that during the competitive period, players had reduced levels of body fat, which affects speed and VO_2max_. Changes in running speed in the middle of the season were also observed by Wislof et al. [15], who found that during the season, the level of sprinting ability of adult players was 1.82 ± 0.3 s for 10 m, 3.0 ± 0.3 s for 20 m, and 4.0 ± 0.2 s for 30 m.

Biological maturation exercises are an important influence on speed, power and strength in youth categories. Wilmore and Costill [28] suggested that the expression of strength in childhood and adolescence relies upon the myelination of motor nerves and neural maturation that is not complete until sexual maturity is reached. Muscle strength in lower limbs increases up to 50% between the 11th and 15th year in boys. The most progressive increase occurs between the 12th and 14th year [29]. We ascribe an important role in improving the speed of these groups by using specific exercises to improve coordination and running technique. A frequent phenomenon in the training process of this age group is the use of a variety of cross-country competitions, which can result in stimulation and subsequent improvement in speed. 

One of the major differences in agility compared to linear speed is that the player learns to anticipate the next move [30], which is an essential part of quality performance during a soccer match. Caldwell and Peters [20] reported significant improvement in the ability of quick change of direction speed at the end of a training period (t_2_ = 14.76 ± 0.38 s), compared to the beginning (t_1_ = 14.97 ± 0.38 s). This is also confirmed by our research, where players at the end of the pre-season training period (t_2_ = 18.52 ± 0.63 s) showed significantly better results than at the beginning (t_1_ = 18.82 ± 0.56 s). The agility 505 test from the pre-season training period was significant only when changing direction towards the non-dominant lower limb (t_1_ = 2.83 ± 0.09 s, t_2_ = 2.76 s). Turning towards the dominant lower limb showed a significant improvement at t_3_ (2.71 ± 0.08 s) and t_4_ (2.72 ± 0.06 s), compared to t_1_ (2.81 ± 0.09 s). Significant improvement (*p* < 0.01) in the test of repeated quick changes of direction speed (IAT) for the pre-season period (t_1_ = 16.5 ± 0.4 s vs. t_2_ = 16.0 ± 0.4 s) was presented by researchers [31], who attributed these changes to the use of small games (5 vs. 5) in the training process. This involved changing direction approximately every three seconds, which may have an impact on improving these skills during the training process. With respect to the fact that in our country, the U13 category plays games on a reduced size field, it can be assumed that these conditions may have an impact on improving acceleration and motor skills that are an essential component of the ability to quickly change direction.

We found that the pre-season period had the highest effect on players’ endurance (Yo-Yo IRT1) where we recorded an increase in players’ performance by 27.2% (p < 0.01). We attribute this to the use of interval games in the training process and the competitiveness of players who, after initial measurements, wanted to provide improved performance and be the best of the team. Casajús [18] reported an increase in VO_2max_ through an incremental running test on a treadmill in adult players in a top Spanish league since the start of the competitive period (50.2 ± 8.0 mL·kg^−1^·min^−1^) to the middle of the season (52.7 ± 8.5 mL·kg^−1^·min^−1^), but this difference was not significant. According to him, the players probably reached their VO_2max_ early in the season, and it was therefore difficult to raise this level further during the season. Achieving such a high level at the beginning of the season could be due to high aerobic-oriented pre-season training, which has been created in order to improve the aerobic component of players. In other studies [6,19], an increased aerobic endurance level at the beginning of the season to the middle of the season was reported, which then decreased, though it was not significant. Wislof et al. [15] identified, through laboratory tests in adult players of the highest level league in Norway, VO_2max_ values of 65.7 ± 4.3 mL·kg^−1^·min^−1^ during the season. Bekris et al. [17] followed changes in the VO_2max_ of adult players from the top Greek league through measurements that were made at the beginning of the pre-season period (t_1_), at the beginning of the competitive period (t_2_), at the end of the first half of the competitive period (t_3_) and after the last round of the competitive period (t_4_). Significant differences in values of VO_2max_ were found when comparing the values at t_2_ (57.16 ± 3.17 mL·kg^−1^·min^−1^) to t_1_ (55.56 ± 3.81 mL·kg^−1^·min^−1^) and t_4_ (56.07 ± 2.84 mL·kg^−1^·min^−1^), and a significant difference was also observed when comparing the values of VO2 max at t_3_ (57.96 ± 2.8 mL·kg^−1^·min^−1^) to t_1_ (55.56 ± 3.81 mL·kg^−1^·min^−1^). VO_2max_ values in the first half of the season increased, but at the end of the season were significantly lower. In our research, during the season (t_3_) we observed further improvement in endurance by 9.9%. A partial drop, however not significant, was observed in our study while comparing the measurements in the middle of the season (t_3_), and at the end of the competitive period (t_4_), which could have been affected by the reduced number of matches at the end of the season.

## 5. Conclusions

The results of this study showed the impact of training and match load at different stages of the training cycle for linear running speed, changes of direction speed and endurance in young soccer players. These results may serve as an indicative basis for comparison with peers in similar research studies. We observed that the performance of speed and the ability to quickly change direction of the players improved throughout the observation period. For endurance, we observed a major improvement during the pre-season training period, while at the end of the season, endurance slightly fell. We conclude that the training process in youth plays an important role in the development of young players and positively affects the development of individual components of athletic performance. The study may help sports practice for clinicians, conditioning coaches, soccer coaches, and physiotherapy coaches by comparing these results to other groups.

## Figures and Tables

**Table 1:** Comparison of physical performance parameters over the season.

Variable		Descriptive Statistic				RM ANOVA			Bonferroni’s Post Hoc Test (*p* < 0.05)
		Mean	SD	95% CI interval		F	*p*	*η_p_^2^*	
				Lower	Upper				
Agility 505DL (s)	t_1_	2.81	0.09	2.77	2.86	6.7	0.001	0.59	t_1_ vs. t_3_, t_4_
	t_2_	2.79	0.13	2.73	2.85				
	t_3_	2.71	0.08	2.67	2.75				
	t_4_	2.72	0.06	2.69	2.75				
Agility 505NL (s)	t_1_	2.83	0.09	2.79	2.88	13.13	0.000	0.43	t_1_ vs. t_2_, t_3_, t_4_ t_2_ vs. t_3_
	t_2_	2.76	0.09	2.71	2.8				
	t_3_	2.7	0.07	2.67	2.74				
	t_4_	2.71	0.09	2.67	2.76				
Ilinois agility test (s)	t_1_	18.82	0.56	18.55	19.09	43.55	0.000	0.71	t_1_ vs. t_2_, t_3_, t_4_ t_2_ vs. t_3_, t_4_
	t_2_	18.52	0.63	18.12	18.82				
	t_3_	17.94	0.51	17.69	18.18				
	t_4_	17.89	0.66	17.57	18.21				
Linear running speed over 30 m (s)	t_1_	5.15	0.21	5.05	5.26	15.47	0.000	0.46	t_1_ vs. t_3_, t_4_ t_2_ vs. t_3_, t_4_
	t_2_	5.07	0.14	5	5.13				
	t_3_	4.99	0.15	4.92	5.07				
	t_4_	4.98	0.17	4.9	5.06				
Yo-Yo IRT1 (m)	t_1_	625.26	170.34	543.16	707.36	48.46	0.000	0.73	t_1_ vs. t_2_, t_3_, t_4_ t_2_ vs. t_3_
	t_2_	858.95	210.55	757.47	960.43				
	t_3_	953.68	229.88	842.88	1064.49				
	t_4_	924.21	215.78	820.21	1028.21				

Notes: t_1_—beginning of the pre-season training period; t_2_—start of the competitive period; t_3_—middle of the competitive period; t_4_—end of the competitive period; SD—standard deviation; CI—confidence interval.

## References

[1] Bloomfield J., Polman R., O’Donoghue P. (2007). Physical demands of different positions in FA Premier League soccer. J. Sports Sci. Med..

[2] Helgerud J., Engen L.C., Wisloff U., Hoff J. (2001). Aerobic endurance training improves soccer performance. Med. Sci. Sports Exerc..

[3] Krustrup P., Mohr M., Ellingsgaard H., Bangsbo J. (2005). Physical demands during an elite female soccer game: Importance of training status. Med. Sci. Sports Exerc..

[4] Hoff J. (2005). Training and testing physical capacities for elite soccer players. J. Sports Sci..

[5] Impellizzeri F.M., Rampinini E., Marcora S.M. (2005). Physiological assessment of aerobic training in soccer. J. Sports Sci..

[6] Bangsbo J. (1993). The physiology of soccer—With special reference to intense intermittent exercise. Acta Physiol. Scand. Suppl..

[7] Svensson M., Drust B. (2005). Testing soccer players. J. Sports Sci..

[8] Bangsbo J., Iaia F.M., Krustrup P. (2007). Metabolic response and fatigue in soccer. Int. J. Sports Physiol. Perform..

[9] Gambetta V. (1996). In a blur: How to develop sport-specific speed. Sports Coach.

[10] Little T., Williams A.G. (2005). Specificity of acceleration, maximum speed, and agility in professional soccer players. J. Strength Cond. Res..

[11] Mohr M., Krustrup P., Bangsbo J. (2003). Match performance of high-standard soccer players with special reference to development of fatigue. J. Sports Sci..

[12] Arnason A., Sigurdsson S.B., Gudmundsson A., Holme I., Engebretsen L., Bahr R. (2004). Physical fitness, injuries, and team performance in soccer. Med. Sci. Sports Exerc..

[13] Wisloeff U., Helgerud J., Hoff J. (1998). Strength and endurance of elite soccer players. Med. Sci. Sports Exerc..

[14] Maly T., Zahalka F., Mala L., Teplan J. (2014). Profile, correlation and structure of speed in youth elite soccer players. J. Hum. Kinet..

[15] Wisløff U., Castagna C., Helgerud J., Jones R., Hoff J. (2004). Strong correlation of maximal squat strength with sprint performance and vertical jump height in elite soccer players. Br. J. Sports Med..

[16] Aziz A.R., Newton M.J., Tan H., Teh K. (2006). Variation in fitness attributes of players during a competitive season in an asian professional soccer league: A field-based investigation. Asian J. Exerc. Sports Sci..

[17] Bekris E., Mylonis L., Gioldasis A., Gissis I., Kombodieta N. (2016). Aerobic and anaerobic capacity of professional soccer players in annual macrocycle. J. Phys. Edu. Sport.

[18] Casajús J.A. (2001). Seasonal variation in fitness variables in professional soccer players. J. Sports Med. Phys. Fit..

[19] Haritonidis K., Koutlianos N., Kouidi E., Haritonidou M., Deligiannis A. (2004). Seasonal variation of aerobic capacity in elite soccer, basketball and volleyball players. J. Hum. Mov. Studies.

[20] Caldwell B.P., Peters D.M. (2009). Seasonal variation in physiological fitness of a semiprofessional soccer team. J. Strength Cond. Res..

[21] Buchheit M., Mendez-Villanueva A., Delhomel G., Brughelli M., Ahmaidi S. (2010). Improving repeated sprint ability in young elite soccer players: Repeated shuttle sprints vs. Explosive strength training. J. Strength Cond. Res..

[22] Harriss D.J., Atkinson G. (2015). Ethical standards in sport and exercise science research: 2016 update. Int. J. Sports Med..

[23] Roozen M. (2004). Illinois agility test. NSCA’s Perform. Train. J..

[24] Bangsbo J., Iaia F.M., Krustrup P. (2008). The Yo-Yo intermittent recovery test. Sports Med..

[25] Cohen J. (1992). A power primer. Psychol. Bull..

[26] Sienkiewicz-Dianzenza E., Rusin M., Stupnicki R. (2009). Anaerobic resistance of soccer players. Fit. Perform. J..

[27] Ostojic S.M. (2003). Seasonal alterations in body composition and sprint performance of elite soccer players. J. Exerc. Physiol..

[28] Wilmore J.H., Costill D.L. (1994). Physiology of Sport and Exercise.

[29] Degache F., Richard R., Edouard P., Oullion R., Calmels P. (2010). The relationship between muscle strength and physiological age: A cross-sectional study in boys aged from 11 to 15. Ann. Phys. Rehabil. Med..

[30] Pearson A. (2001). Speed, Agility and Quickness for Soccer: Saq Soccer.

[31] Mercer T., Gleeson N., Mitchell J. (1997). Fitness profiles of professional soccer players before and after pre-season conditioning. Sci. Footb..

